# Dietary methionine restriction attenuates renal ischaemia/reperfusion‐induced myocardial injury by activating the CSE/H2S/ERS pathway in diabetic mice

**DOI:** 10.1111/jcmm.15578

**Published:** 2020-08-13

**Authors:** Yuanyuan Pan, Minghuan Fu, Xiaohan Chen, Jing Guo, Biao Chen, Xuefei Tao

**Affiliations:** ^1^ Department of Gerontology Sichuan Academy of Medical Sciences & Sichuan Provincial People’s Hospital Chengdu China; ^2^ Department of Cardiac Surgery Sichuan Academy of Medical Sciences & Sichuan Provincial People’s Hospital Chengdu China

**Keywords:** apoptosis, CSE, dietary methionine restriction, ERS, hydrogen disulphide, ischaemia, reperfusion

## Abstract

Methionine restrictive diet may alleviate ischaemia/reperfusion (I/R)‐induced myocardial injury, but its underlying mechanism remains unclear. HE staining was performed to evaluate the myocardial injury caused by I/R and the effect of methionine‐restricted diet (MRD) in I/R mice. IHC and Western blot were carried out to analyse the expression of CSE, CHOP and active caspase3 in I/R mice and hypoxia/reoxygenation (H/R) cells. TUNEL assay and flow cytometry were used to assess the apoptotic status of I/R mice and H/R cells. MTT was performed to analyse the proliferation of H/R cells. H2S assay was used to evaluate the concentration of H2S in the myocardial tissues and peripheral blood of I/R mice. I/R‐induced mediated myocardial injury and apoptosis were partially reversed by methionine‐restricted diet (MRD) via the down‐regulation of CSE expression and up‐regulation of CHOP and active caspase3 expression. The decreased H2S concentration in myocardial tissues and peripheral blood of I/R mice was increased by MRD. Accordingly, in a cellular model of I/R injury established with H9C2 cells, cell proliferation was inhibited, cell apoptosis was increased, and the expressions of CSE, CHOP and active caspase3 were dysregulated, whereas NaHS treatment alleviated the effect of I/R injury in H9C2 cells in a dose‐dependent manner. This study provided a deep insight into the mechanism underlying the role of MRD in I/R‐induced myocardial injury.

## 1 INTRODUCTION

As a severe pathological disorder caused by initial blood supply deficiency and subsequent reoxygenation, ischaemia‐reperfusion (I/R) may lead to hypertension, trauma, sepsis, shock and organ failures including bowel infarct, liver failure and renal tubular necrosis.^1^ I/R may also lead to a wide range of complications in the vascular system including stroke and myocardial infarction.^1^, ^2^ Multiple factors, including complement system activation, endoplasmic reticulum stress, tissue calcium overload, oxidative phosphorylation, free radicals concentration, endothelial dysfunction and the activation of apoptotic, necrotic and autophagic signalling, were all suspected to be underlying pathologic mechanisms of I/R‐induced damages.^1^, ^3^ Interestingly, methionine‐restricted diet (MRD) could decrease body weight, reduce the levels of lipids in the liver and decrease the level of ROS synthesis.^4^ In addition, MRD can trigger the uptake of glucose by elevating the synthesis of hydrogen sulphide (H2S) in mice.^5^ It should be noted that the H2S in mammalian cells is synthesized from L‐cysteine under the catalysis of cystathionine gamma‐lyase (CSE) (also termed as cysteine desulfhydrase as well as cystathionine β‐synthase (CBS)).^6^, ^7^, ^8^, ^9^

Multiple specific CSE and CBS inhibitors have been developed. For example, D, L‐Propargylglycine and β‐cyano‐L‐alanine can both selectively suppress the activity of CSE.^6^, ^9^, ^10^, ^11^ On the other hand, metabolites of L‐cysteine, such as ammonia, H2S, and pyruvate, cannot suppress the activity of CSE.^12^ Interestingly, chelerythrine (CHE) can preserve the functions of remote myocardiocytes after I/R‐caused damages in rats of diabetes via up‐regulating the endogenous expression of CES and H2S while inhibiting the PKC/NF‐κB signalling.^13^

The disruption of homoeostasis in ER of cells can lead to endoplasmic reticulum stress (ERS), which is involved in the pathogenesis of many cardiovascular disorders. The apoptosis of VSMCs in the presence of ERS triggers the phenotypic VSMC transformation, whereas the treatment with H2S can protect cardiovascular tissues against ERS.^14^, ^15^, ^16^, ^17^, ^18^ As I/R can impair ER integrity while increasing the level of ERS, the reduction in ERS may play a protective role in damages induced by myocardial I/R.^19^ Interestingly, ERS can also exert a protective effect to promote myocardiocyte survival in the initial stages of myocardial I/R‐induced damages.^20^, ^21^

It has been shown that dietary methionine restriction promoted the expression of CSE, leading to increased production of H2S.^5^, ^6^, ^7^, ^8^, ^9^ Furthermore, H2S has been reported to suppress the activation of ERS^14^, ^15^, ^16^, ^17^, ^18^ and inactivation of ERS was found to alleviate myocardial ischaemia‐reperfusion injury.^20^, ^21^ Based on these evidences, we hypothesized that dietary methionine restriction may alleviate myocardial ischaemia‐reperfusion injury by regulating CSE/H2S/ERS signalling pathway. In this study, we observed the effect of MRD on the myocardiac I/R as well as its effect on the signalling pathways of CSE/H2S/ERS in mice.

## 2 MATERIALS AND METHODS

### 2.1 Animals

All experimental procedures in this study were performed in strict accordance with an approved animal study protocol reviewed by our Animal Ethics Committee. In this study, an I/R mouse model was established as previously described (10) and their myocardial tissues were harvested for HE staining to evaluate the extent of IR injury. In brief, mice were purchased from our animal centre and divided into four groups: 1. SHAM (sham‐operated mice); 2. SHAM + MRD (sham‐operated mice receiving a methionine restrictive diet); 3. I/R (ischaemia/reperfusion mice); and 4. I/R + MRD (ischaemia/reperfusion mice receiving a methionine restrictive diet). Accordingly, the ‘MRD’ treatment indicated that a diet starting from 24 hours after the completion of ligation with a 0.17% methionine added.^25^ Moreover, to establish the I/R model, the mice were placed under anaesthesia by using 200 g per kg of medetomidine hydrochloride along with 50 mg per kg of ketamine hydrochloride (Merck, Darmstadt, Germany) given via intraperitoneal injection (Merck). Then, the mice were subjected to an endotracheal intubation operation to keep their normal levels of respiratory functions. In the next step, the left anterior descending coronary artery in each mouse was temporarily ligated to the left atrium and conus artery by using a prolene suture with a size of 6‐0. After the colour in the apex and anterior ventricular wall of the heart was changed, which indicated successful ischaemia, the mouse was kept for 30 minutes under the ischaemic condition and then re‐perfused for 24 hours. All animal experiments were performed in line with the Guide for the Care and Use of Laboratory Animal by International Committees and were approved by the Institutional Animal Ethics Committee.

### 2.2 Cell culture and cell treatment

H9C2 cells were acquired from ATCC and maintained in a modified DMEM (Invitrogen, Carlsbad, CA) added with 10% FBS and appropriate antibiotics (Thermo Fisher Scientific, Waltham, MA). Then, the cells were divided into 4 groups, that is 1. Control; 2. H/R; 3. hypoxia/reoxygenation (H/R) + NaHS (100 µM); and 4. H/R + NaHS (400 µM). The cells in groups 2‐4 were first subjected to H/R treatment to establish a cell model of I/R‐induced damages. In groups 3 and 4, different concentrations of NaHS were added to H9C2 cells after the cell model of I/R‐induced damages was successfully established.^26^

### 2.3 H/R treatment of H9C2 cells

To establish a cell model of I/R‐induced damages, H9C2 cells were placed in a hypoxic incubator containing 94% of N2, 5% of CO2 and 1% of O2. In addition, the cells were maintained in a D‐Hank solution containing 5.37 mmol/L of KCl, 0.44 mmol/L of KH2PO4, 136.89 mmol/L of NaCl, 4.166 mmol/L of NaHCO3, 0.338 mmol/L of Na2HPO4 and 5 mmol/L of d‐glucose. The pH of the D‐Hank solution was between 7.3 and 7.4. After the H9C2 cells were cultured at 37°C under the hypoxic condition for 24 hours, they were transferred into a fresh DMEM for subsequent assays. For cells undergoing NaHS treatments, the H/R cells were treated by 100 or 400 µmol/L of NaHS for 30 minutes before subsequent assays.^27^

### 2.4 Cell proliferation assay

To analysis the proliferation status of cells, a CCK‐8 assay kit (Dojindo Molecular Technologies, Kumamoto, Japan) was used following the manual of the experimental kit.

### 2.5 Western blot analysis

Collected tissue and cell samples were first subjected to lysis in a pH 7.5 lysis buffer containing 50 mmol/L of Tris, 150 mmol/L of NaCl, 5 mmol/L of MgCl_2_, 10% of glycerol, 1 mm of DTT, 1 mm of EDTA, 0.5% of Triton X‐100 and a mixture of phosphatase, protease and asphosphatase inhibitors (Sigma Aldrich, St. Louis, MO). After the concentration of protein in the lysate was measured by utilizing a BCA assay (Bio‐Rad, Hercules, CA) following kit instruction, the sample protein was resolved by 10% SDS‐PAGE, blotted onto PVDF membranes, incubated with primary anti‐CSE, anti‐CHOP and anti‐active caspase‐3 antibodies at 4 degree for 24 hours, washed and incubated for 2 hours at room temperature with corresponding secondary antibodies (all antibodies were acquired from Abcam, Cambridge, MA). After staining the membrane with ECL kit (Sigma Aldrich, St. Louis, MO), the relative protein expression of CSE, CHOP and active caspase‐3 was calculated.

### 2.6 Apoptosis analysis

The apoptotic profiles of collected samples were analysed by using an Annexin V‐FITC/PI staining kit (Thermo Fisher Scientific, Waltham, MA) following the manufacturer's instructions. After the samples were stained using the reagents provided in the kit, they were fixed in 70% ethanol at 4 degree and then loaded onto a BD FACSCalibur flow cytometer (BD, San Jose, CA), and the apoptotic profiles of the samples were analysed by using CellQuest software.

### 2.7 Immunohistochemistry (IHC) assay

In the IHC assay, the samples were initially fixed for 15 minutes at room temperature by using PBS containing 4% paraformaldehyde. Then, the sample slides were blocked by 10% goat serum (Thermo Fisher Scientific, Waltham, MA), permeabilized at room temperature for 1 hour by using 0.1% Triton X‐100 (Sigma Aldrich, St. Louis, MO), incubated at 4 degrees for 24 hours with primary anti‐CSE antibodies (Abcam, Cambridge, MA), washed with PBS, incubated for another 2 hours at room temperature with appropriate biotin‐conjugated secondary antibodies (Abcam, Cambridge, MA), stained with DAPI (Sigma Aldrich, St. Louis, MO), counterstained with haematoxylin and finally observed underneath an Olympus IX71 microscope (Olympus, Tokyo, Japan) to evaluate the positive protein expression of CSE in the samples using Prism 8.0 software (GraphPad, San Diego, CA).

### 2.8 H&E assay

The level of I/R‐induced injury of mouse cardiac tissues was evaluated using an H&E assay (Thermo Fisher Scientific, Waltham, MA) following the method suggested by the kit manufacturer. In brief, the tissues were first fixed using PBS containing 4% paraformaldehyde (Sigma Aldrich, St. Louis, MO), sliced into 4 um sections, treated with 3% H2O2 to block the activity of endogenous peroxide, further treated with 10% BSA to block non‐specific binding and then stained with reagents in the H&E assay kit to evaluate the extent of I/R‐induced injury in each sample.

### 2.9 Assay of levels of H2S, CSE and CBS

The levels of endogenous H2S and the activities of CSE and CBS in collected plasma and cardiac tissue samples were measured by using corresponding commercially available assay kits (Huijia Bioengineering, Xiamen, China) in accordance with the assay procedures provide by the manufacturer.

### 2.10 Terminal deoxynucleotidyl transferase (TdT) mediated dUTP nick‐end labelling (TUNEL) assay

A TUNEL experimental kit (Boster, Wuhan, China) was utilized to evaluate the apoptotic profiles of collected samples following the kit instruction.

### 2.11 Statistical analysis

One‐way ANOVA and Student's *t* tests were utilized to compare differences among different groups. All experimental results were shown as mean ± SD. The *P* value for the level of statistical significance was set to .05.

## 3 RESULTS

### 3.1 I/R‐caused myocardial dysfunction MRD could partially reverse I/R‐induced dysfunction

**Figure 1:**
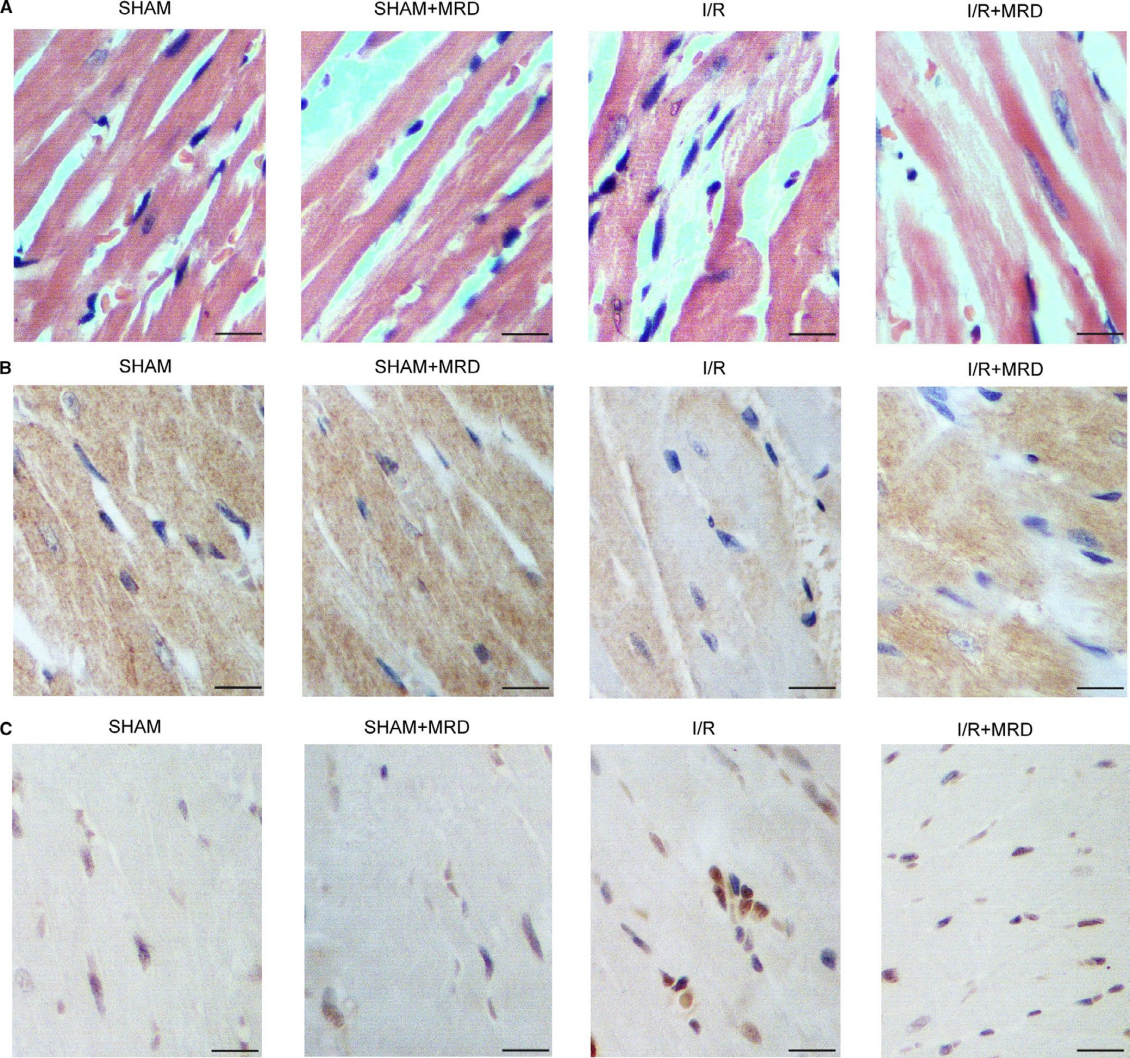
MRD partially reversed I/R‐induced myocardial dysfunction. A,HE staining showed that I/R increased the level of myocardial injury, whereas MRD reduced the level of injury caused by I/R. B, IHC results showed that CSE expression was elevated in the myocardial tissues of I/R mice, whereas MRD reduced the level of CSE expression in I/R mice. C, TUNEL analysis indicated that myocardial apoptosis was significantly promoted by I/R, and MRD reduced the apoptosis in I/R mice to a certain degree

An I/R mice model was established as previously described, and their myocardial tissues were harvested for HE staining to evaluate the extent of I/R injury. Myocardial injury was the most severe in I/R mice, whereas MRD considerably reduced the extent of I/R injury (Figure 1A). Immunohistochemistry was performed to analyse the differential expression of CSE in the myocardial tissues of I/R and control mice. I/R resulted in a dramatic decrease of CSE expression, whereas MRD partly restored the expression of CSE (Figure 1B). TUNEL assay was carried out to assess the apoptotic status of myocardial tissues in I/R mice, which showed a remarkably elevated level of apoptosis, although MRD alleviated the extent of apoptosis caused by I/R (Figure 1C).

### 3.2 MRD partially restored the normal levels of H2S, CSE, CHOP and active caspase3 in I/R mice

**Figure 2:**
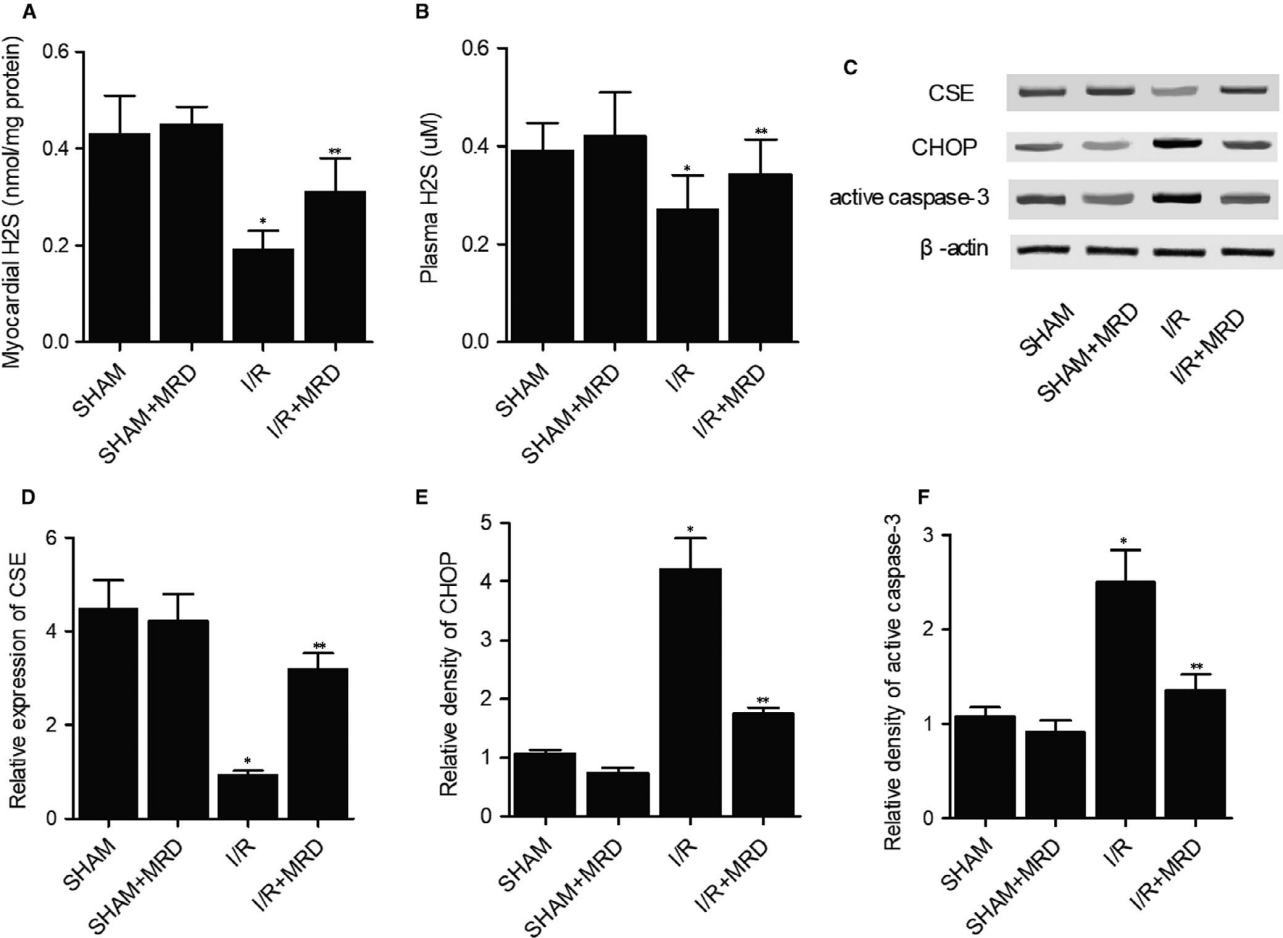
MRD restored the aberrant expression of H2S, CSE, CHOP and active caspase3 in I/R in mice (*P value < .05 compared with SHAM group; **P value < .05 compared with I/R group). A, Concentration of H2S in the myocardial tissues was decreased in I/R mice and elevated by MRD. B, Concentration of H2S in the peripheral blood was declined in I/R mice and increased by MRD. C, Differential expression of CSE, CHOP and active caspase3 in I/R mice. D, Expression of CSE was inhibited in I/R mice and restored by MRD. E, Expression of CHOP was increased in I/R mice and decreased by MRD. F, Expression of CHOP was enhanced in I/R mice and reduced by MRD

As H2S signalling pathway is closely correlated with the extent of I/R injury, H2S concentration was analysed in the myocardial tissues (Figure 2A) and peripheral blood (Figure 2B) of I/R mice. As shown in Figure 2, H2S concentration was notably decreased in the myocardial tissues and peripheral blood of I/R mice, whereas MRD obviously restored the concentration of H2S in both myocardial tissues and peripheral blood. Then, the expression of CSE (Figure 2C and D), CHOP (Figure 2C and E) and active caspase‐3 (Figure 2C and F) was analysed using Western blot. The CSE expression in I/R mice was dramatically reduced, whereas MRD restored the expression of CSE in I/R mice. On the contrary, the expression of CHOP and active caspase3 was evidently promoted in I/R mice, whereas MRD suppressed the expression of CHOP and active caspase3 I/R mice to a certain extent.

### 3.3 Hypoxia/reoxygenation reduced cell proliferation and increased apoptosis, whereas NaHS reduced the effect of hypoxia/reoxygenation in a dose‐dependent manner

**Figure 3:**
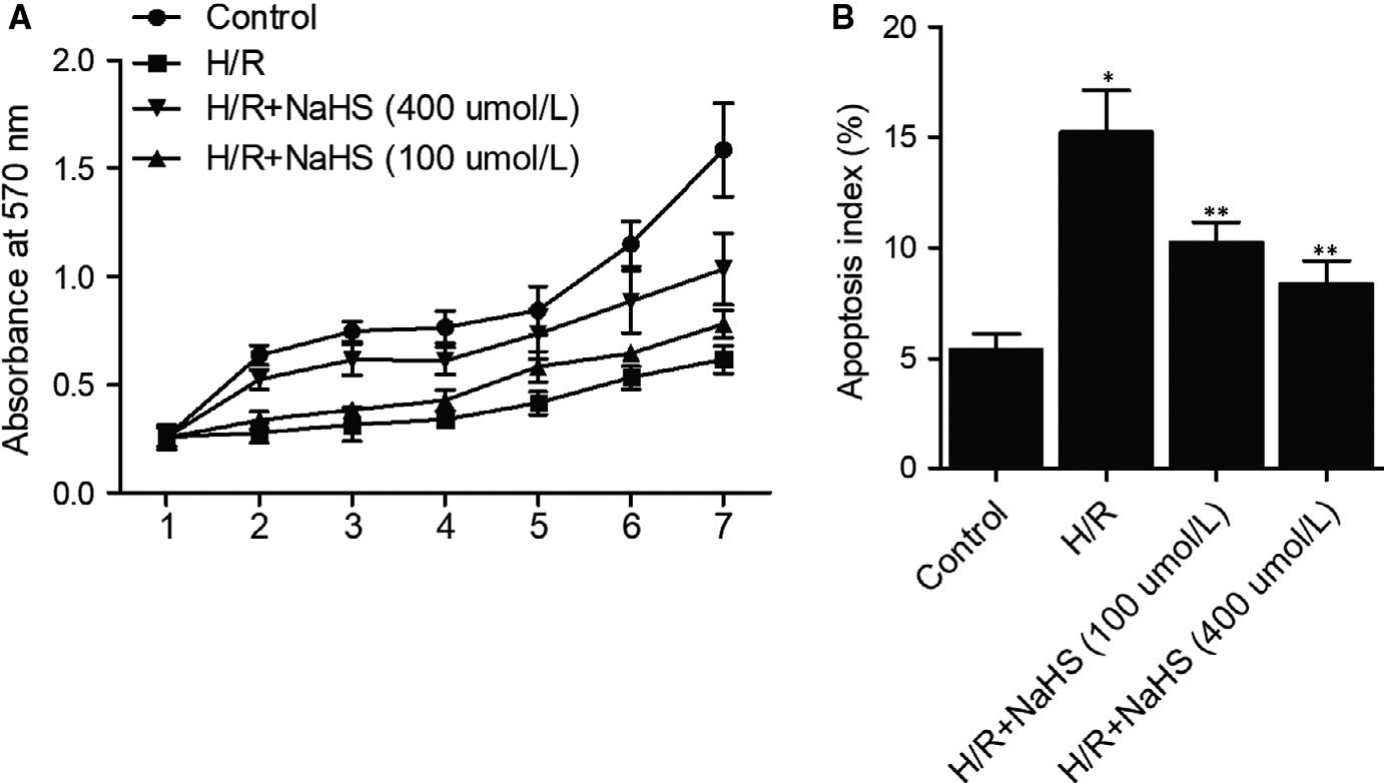
Hypoxia/reoxygenation reduced cell proliferation and promoted apoptosis, whereas NaHS reduced the effect of hypoxia/reoxygenation in a dose‐dependent manner in H9C2 cells (*P value < .05 compared with control group; **P value < .05 compared with HR group). A, Proliferation of H9C2 cells was reduced under H/R and increased by NaHS in a concentration‐dependent fashion. B, Apoptosis of H9C2 cells was dramatically increased under H/R and reduced by NaHS in a dose‐dependent manner

H9C2 cells were subjected to hypoxia/reoxygenation (H/R) to construct a cellular model of I/R injury. NaHS was then added to I/R cells to evaluate its effect on I/R cell proliferation and apoptosis. The proliferation of H9C2 cells was suppressed by H/R effectively, whereas NaHS significantly restored the proliferation of I/R cells in a dose‐dependent manner (Figure 3A). At the same time, flow cytometry analysis showed that the apoptosis index of I/R cells was dramatically increased, whereas NaHS significantly reduced the apoptosis index of I/R cells in a dose‐dependent manner (Figure 3B).

### 3.4 NaHS treatment restored the dysregulated expression of CSE, CHOP and active caspase3 in I/R cells

**Figure 4:**
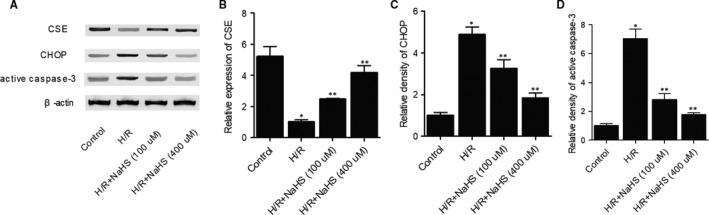
NaHS treatment dose‐dependently restored the dysregulated expression of CSE, CHOP and active caspase3 in H9C2 cells under hypoxia/reoxygenation (*P value < .05 compared with control group; **P value < .05 compared with HR group). A, Differential expression of CSE, CHOP and active caspase3 in H9C2 cells under H/R and different concentrations of NaHS. B, Expression of CSE was inhibited in H/R cells but restored by NaHS treatment in a dose‐dependent manner. C, Expression of CHOP was enhanced in H/R cells but reduced by NaHS treatment in a dose‐dependent manner. D, Expression of CHOP was enhanced in H/R cells but reduced by NaHS treatment in a dose‐dependent manner

Furthermore, the expression of CSE (Figure 4A and B), CHOP (Figure 4A and C) and active caspase3 (Figure 4A and D) was analysed in H9C2 cells treated under different conditions. H/R dramatically suppressed the expression of CSE, whereas NaHS significantly increased the expression of CSE in I/R cells in a dose‐dependent manner. On the opposite site, the expression of CHOP and active caspase3 was dramatically enhanced in I/R cells, whereas NaHS significantly decreased the expression of CSE in I/R cells in a dose‐dependent manner (Figure 4).

## 4 DISCUSSION

Methionine‐restricted diet (MRD) can increase human lifespan by improving body metabolism.^28^ Similar effects of MRD were observed in other species such as nematodes, drosophila, rodents and yeast.^29^, ^30^, ^31^, ^32^ In this study, we established an I/R mice model and performed HE staining to show that I/R‐induced myocardial injury could be partially alleviated by MRD. Furthermore, we carried out a TUNEL assay to analyse the apoptotic status of myocardial tissues in I/R mice and showed that cell apoptosis was significantly elevated by I/R and reduced by MRD.

MRD was found to enhance the activity of CSE in the liver and to promote the endogenous synthesis of H2S in mice given a normal diet.^33^ Nevertheless, the mechanisms underlying the beneficial effect of MRD in the treatment of I/R‐induced injury remain unclear.^34^ Fasting seems to reduce the damages of the vasculature caused by intimal hyperplasia.^35^ In addition, fasting can prevent I/R‐induced brain damages in rats suffering from cerebral artery occlusion.^33^, ^36^ In this study, we performed IHC and Western blot to detect the differential expression of CSE, CHOP and active caspase3 in mice and cells of different groups. CSE expression was suppressed whereas the expression of CHOP and active caspase3 was remarkably increased by I/R and H/R. In contrary, MRD and NaHS reversed the effects of/R and H/R treatments.

Produced endogenously in the liver, H2S is implicated in a wide range of physiological processes by regulating the functions of mitochondria.^37^, ^38^, ^39^, ^40^ When the levels of oxygen in cells change, the CSE present in the cytosol enters mitochondria to enhance the synthesis of H2S, subsequently keeping a normal level of ATP synthesis under hypoxic condition.^41^ H2S also plays a protective role in I/R‐induced damages in mouse brain by promoting cerebral vasodilatation.^42^ In fact, there is a direct correlation between the level of H2S synthesis in the brain and the extent of cerebrovascular relaxation. In this study, we evaluated the concentration of H2S in the myocardial tissues and peripheral blood of I/R mice, and showed that MRD recovered the concentration of H2S reduced in I/R mice.

H2S has been deemed as an important signalling factor with an essential role in the cardiovascular system.^43^ For example, H2S can be produced from L‐cysteine in blood vessels, myocardium and fibroblasts in the heart under the functions of 3‐MST, CBS and CSE to exert a protective effect on cardiovascular tissues during I/R.^44^

A previous study demonstrated that H2S inhibited the apoptosis of cardiomyocytes caused by myocardial ischaemia/reperfusion (MIR) and H/R.^45^ In addition, an increased H2S level in serum samples collected from rats suffering from I/R‐induced damages can be attributed to the generation of self‐protective responses upon the onset of I/R.^24^, ^46^

Past reports demonstrated that H2S can suppress the apoptosis of epithelial cells in the bronchial tubes by reducing ERS. Recently, a similar study suggested that H2S can suppress the apoptosis of neurons mediated by ERS in the hippocampus of rats.^47^ In addition, H2S can protect myocardial tissues against I/R‐induced damages in rats by inhibiting the generation of ERS.^48^ In another study, the mechanism underlying the role of ERS in cell apoptosis after the onset of I/R damages was discussed, although little is known about the specific factors involved in such mechanisms.^27^ In this study, MTT and flow cytometry were performed to evaluate the proliferation and apoptosis of H9C2 cells treated with H/R and NaHS. We found that NaHS helped to increase the proliferation while reducing the apoptosis of I/R cells in a dose‐dependent manner. In a recent study, the authors showed that ERS plays a critical role in promoting the apoptosis of multiple types of cells after the onset of I/R‐induced damages in mice. Furthermore, a moderate level of ERS present in the initial stage of stress can reduce cell apoptosis.^49^ Therefore, there is a close correlation between the level of ERS and the apoptosis of myocardiocytes. Other studies demonstrated that an excessive level of ERS can be induced by certain factors, including ischaemia and hypoxia, which subsequently induces the death of myocardiocytes via the signalling pathways of JNK, caspase‐12 and CHOP.^22^, ^23^, ^50^

## 5 CONCLUSION

The results of the present study revealed a key role of MRD in the regulation of oxidative stress generated during I/R. Endogenous H2S also helps to reduce the severity of diabetic RI/RMI by inhibiting the H2S/ERS pathway. MRD could promote the expression of endogenous CSE/H2S and may be considered as a therapeutic target in the treatment of diabetic RI/RMI.

## CONFLICT OF INTEREST

None.

## AUTHOR CONTRIBUTION

**Yuanyuan Pan:** Conceptualization (equal); Formal analysis (equal); Investigation (equal). **Minghuan Fu:** Investigation (equal); Methodology (equal). **Xiaohan Chen:** Investigation (equal); Validation (equal). **Jing Guo:** Investigation (equal); Software (equal); Supervision (equal); Writing‐original draft (equal). **Biao Chen:** Validation (equal); Writing‐original draft (equal). **Xuefei Tao:** Investigation (equal); Writing‐original draft (equal); Writing‐review & editing (equal).

## Data Availability

The data that support the findings of this study are available from the corresponding author upon reasonable request.
